# Running into trouble: exercise-exacerbated recurrent pericarditis—a case report

**DOI:** 10.1093/ehjcr/ytaf243

**Published:** 2025-05-16

**Authors:** Tess Calcagno, Jibran Ikram, Felix Berglund, Allan Klein

**Affiliations:** Department of Cardiovascular Medicine, Cleveland Clinic Foundation, 3566 Grosvenor Rd, Cleveland Heights, OH 44118, USA; Department of Cardiovascular Medicine, Cleveland Clinic Foundation, 3566 Grosvenor Rd, Cleveland Heights, OH 44118, USA; Department of Cardiovascular Medicine, Cleveland Clinic Foundation, 3566 Grosvenor Rd, Cleveland Heights, OH 44118, USA; Department of Cardiovascular Medicine, Cleveland Clinic Foundation, 3566 Grosvenor Rd, Cleveland Heights, OH 44118, USA

**Keywords:** Case report, Pericarditis, Exercise, Cardiac MRI, Inflammation, Biologics, Imaging, LGE

## Abstract

**Background:**

Recurrent pericarditis is an inflammatory condition characterized by symptom recurrence after an initial episode. Exercise-induced flares are underrecognized.

**Case Summary:**

A 53-year-old male presented with recurrent pericarditis, initially diagnosed in June 2020 and inadequately treated with a three-month colchicine course. He experienced four exercise-induced flares coinciding with triathlon training, each resolving spontaneously. In August 2024, the patient presented with severe right shoulder pain and tachycardia. Diagnostic evaluation revealed a moderate pericardial effusion, prompting a pericardial window. Despite colchicine and NSAIDs, symptoms recurred, and MRI confirmed active pericarditis with late gadolinium enhancement of the pericardium. Given the refractory nature of his disease, biologic therapy with rilonacept was initiated.

**Discussion:**

This case highlights the association between high-intensity exercise and recurrent pericarditis, underscoring the need for individualized treatment strategies, including biologics. It contributes to the understanding of refractory cases and exercise-induced inflammation.

**Learning points**

- **Exercise as a Trigger for Recurrent Pericarditis:** High-intensity exercise can exacerbate subclinical pericardial inflammation, leading to recurrent episodes of pericarditis. Identifying and managing such triggers is crucial in preventing recurrence and optimizing treatment outcomes.
- **Role of Biologics in Refractory Pericarditis:** For patients with recurrent pericarditis unresponsive to standard therapies such as colchicine and NSAIDs, biologics like rilonacept targeting the IL-1 inflammatory pathway can be effective in reducing recurrence rates and improving quality of life.

## Introduction

Recurrent pericarditis remains challenging, especially when standard treatments fail, and high-intensity exercise worsens symptoms.^1^ This case of a competitive triathlete with exercise-triggered recurrence highlights the need for individualized care, including biologic therapy. It emphasizes balancing symptom control with lifestyle goals and calls for more evidence to guide safe exercise in refractory cases. This case contributes to the growing understanding of exercise-induced mechanical and immune activation in recurrent pericarditis, offering insights into the role of advanced therapies like rilonacept and their potential to improve quality of life in such complex presentations.

## Summary figure

**Figure:**
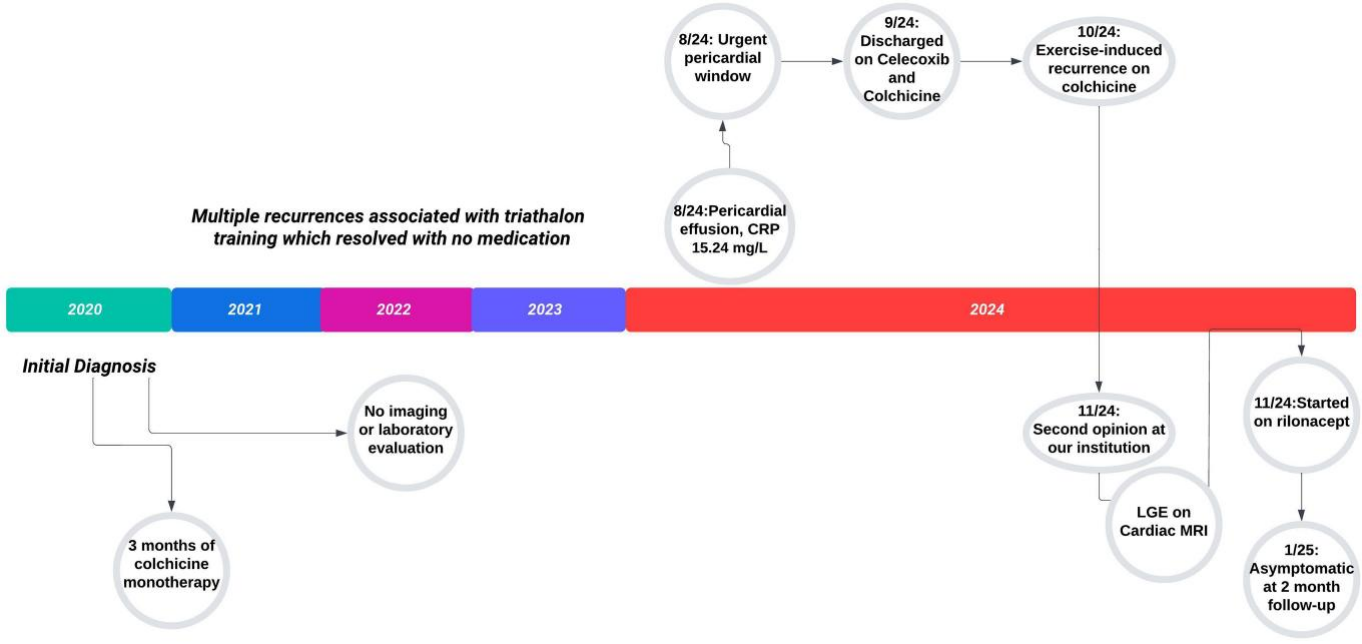


## Case presentation

A 53-year-old male with no significant past medical history presented to our tertiary pericardial referral centre seeking a second opinion regarding chronic management of recurrent pericarditis. At the time of the appointment, he was asymptomatic and on colchicine alone but expressed concerns about his history of recurrent episodes and the impact on his ability to resume triathlon training. The patient reported prior symptoms primarily triggered by high-intensity exercise, including pleuritic chest pain and fatigue. He was eager to explore biologic therapy to prevent future recurrences and allow a return to physical activity.

The patient was a competitive triathlete regularly training for Sprint, Olympic, and Half-Ironman events. His regimen included 3–5 swim sessions (2500–4000 m, 60–90 min), 3–5 cycling workouts (60-min intervals to 3–5-h endurance rides), and 4–6 runs weekly (tempo, long runs up to 2 h, speed intervals). He denied performance-enhancing drug or supplement use aside from whey protein. Exam showed no signs of anabolic steroid use—e.g. acne, gynaecomastia, testicular atrophy, or muscle hypertrophy—and findings aligned with indirect clinical markers proposed in the literature.^2^

The patient was first diagnosed with idiopathic pericarditis in June 2020, presenting with sharp, pleuritic chest pain radiating to his shoulder, which was exacerbated by inspiration. He was treated with a 3-month course of colchicine at 0.6 mg twice a day without follow-up.

At that time, his laboratory evaluation revealed an elevated ESR, as documented in the chart review, but echocardiography showed no significant abnormalities. A viral and autoimmune workup was negative, ruling out infectious or systemic inflammatory aetiologies.

Over the next 4 years, he experienced four distinct recurrences of pleuritic chest pain, each lasting approximately 1 week. These episodes coincided with intense periods of triathlon training, with symptoms characterized by a pattern of recurrence and spontaneous resolution upon tapering down exercise intensity. The patient did not seek medical attention or initiate any specific treatment during these recurrences, as symptoms consistently resolved without intervention.

He was admitted in August 2024 with severe chest pain that began during triathlon training. Imaging revealed a moderate pericardial effusion. After multidisciplinary discussion, the patient elected to undergo a pericardial window procedure to provide more definitive relief and facilitate a timely return to training. He was discharged with 30 days of Celecoxib and colchicine.

**Figure 1:**
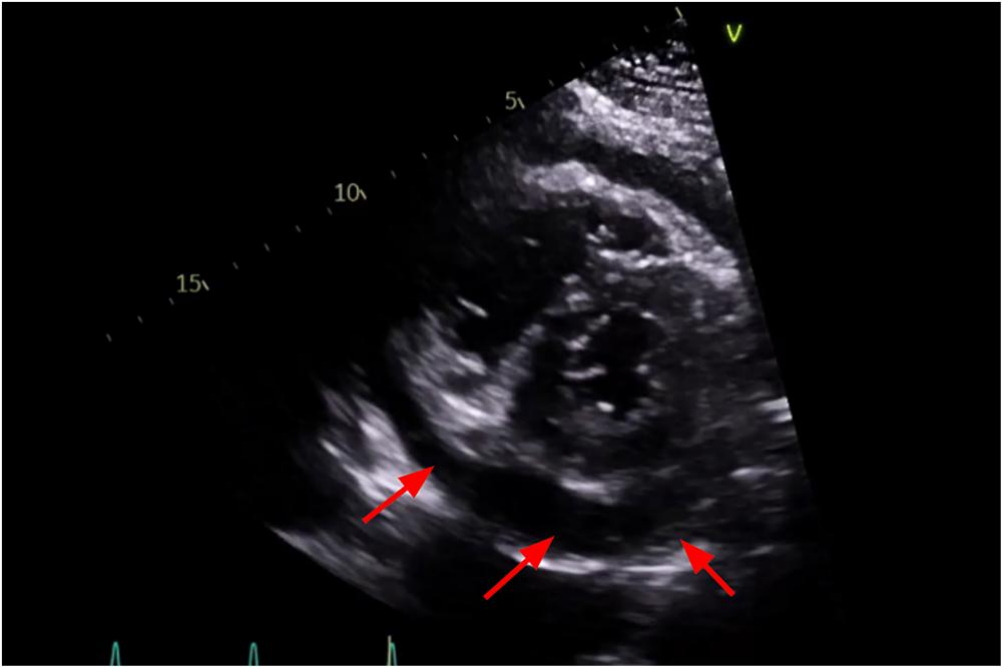
Initial transthoracic echocardiogram. Parasternal short-axis view at the mid-papillary level highlights a pericardial effusion. The anechoic (red arrows) region surrounding the heart, seen most prominently posteriorly, represents fluid accumulation within the pericardial sac. The effusion appears circumferential, with no evidence of tamponade physiology.

**Figure 2:**
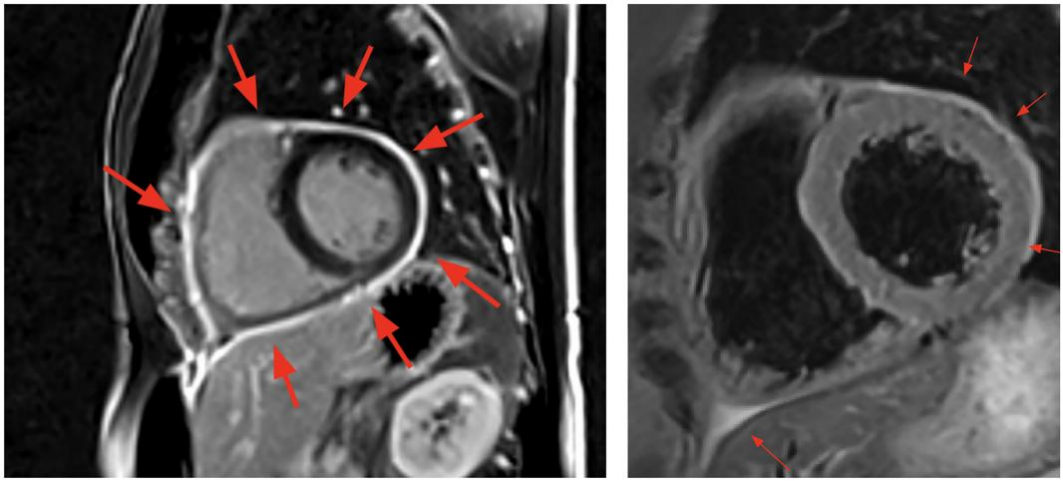
Cardiac MRI with late gadolinium enhancement and T2 Stir. The left panel demonstrates a sagittal late gadolinium enhancement image, highlighting circumferential pericardial delayed enhancement (red arrows), consistent with pericardial inflammation and scar. The right panel displays a T2 STIR image, which reveals pericardial oedema (red arrows).

On examination, he was tachycardic with a pericardial rub noted on auscultation but demonstrated no clinical signs of tamponade. Echocardiography performed in August 2024 revealed a moderate pericardial effusion approximately 15 mm in thickness, right atrial collapse during late diastole, normal left ventricular function with an ejection fraction (EF) of 58%, and mild dilation of the right atrium and right ventricle (*Figure 1*). MRI performed in November of 2024 during our office visit confirmed active pericarditis, showing pericardial oedema on T2 STIR imaging and circumferential late gadolinium enhancement (LGE) showing pericardial inflammation (*Figure 2*). Laboratory findings demonstrated persistent inflammation, with an elevated his high-sensitivity C-reactive protein(hs-CRP) before the pericardial window, which decreased to 8.2 mg/L (normal <3.0 mg/L) post-procedure, and an ESR that decreased from 78 mm/h to 70 mm/h (normal <15 mm/h). An autoimmune panel was negative for lupus, rheumatoid arthritis, ANCA vasculitis, and sarcoidosis. Parvovirus B19 IgG was positive at a titer of 1:40 (enzyme-linked immunosorbent assay, ELISA), consistent with prior exposure but not indicative of active infection.

The patient underwent a pericardial window in August 2024 to relieve symptoms and manage the pericardial effusion. Post-procedure, he was discharged on colchicine (0.6 mg twice daily) and celecoxib (200 mg daily). Due to persistent symptoms, celecoxib was increased to 400 mg daily, and prednisone was initiated in September 2024. Despite these interventions, the patient experienced a flare in October 2024, triggered by increase in running volume characterized by chest pain and fatigue. Given the refractory nature of his disease, biologic therapy with rilonacept was initiated. Pre-treatment laboratory evaluations were completed, and the patient was scheduled for virtual follow-up in 3 months to assess his response. Plans were made for ongoing evaluation of symptoms and inflammatory markers, and the patient expressed optimism about his potential response to rilonacept.

Two weeks after his initial appointment, the patient was started on rilonacept at a loading dose of 320 mg subcutaneously, followed by 160 mg weekly maintenance dosing. He was also prescribed colchicine 0.6 mg daily as adjunctive therapy. Over the following month, the patient reported continued asymptomatic status, with no recurrence of chest discomfort or fatigue. At his one-month follow-up after starting rilonacept, given his stable condition and persistent improvement, colchicine was discontinued.

At 2-month follow-up, labs showed resolution of inflammation (CRP <1 mg/L, normal ESR). The patient reported only mild injection site reactions, managed with topical hydrocortisone. He remained otherwise asymptomatic. Though triathlon training was still restricted, he was allowed low-intensity activity like brisk walking to support reconditioning. Future training progression would be guided by clinical status and ventilatory thresholds on CPET, per expert recommendations.^3^ While frustrated and anxious about delayed return, he remained hopeful. A 6-month follow-up was scheduled to reassess symptoms, review imaging, and discuss a structured return-to-training plan.

Timeline of his treatment course is outlined in the central figure.

## Discussion

This case illustrates the underrecognized association between high-intensity exercise and recurrent pericarditis. Mechanical stress and immune activation are proposed mechanisms by which intense exercise exacerbates subclinical pericardial inflammation, leading to recurrent flares.^4^

Current guidelines advise that non-competitive athletes should limit physical activity until symptoms resolve and biomarkers normalize. Competitive athletes are advised to avoid competitive sports until symptoms subside, biomarkers return to normal, and specific criteria are met, including the absence of fever, no pericardial effusion, and normalized inflammatory markers such as erythrocyte sedimentation rate (ESR) and C-reactive protein (CRP).^5^ The European Association of Preventive Cardiology (EAPC) (2019) recommends avoiding competitive sports during acute pericarditis and delaying sports resumption for 1–3 months after the resolution of the active phase, provided biomarkers normalize, left ventricular function is preserved, and no arrhythmias are detected on 24-hour ECG or exercise testing.^6^ The European Society of Cardiology (ESC) (2020) provides broader guidelines discouraging both competitive sports and leisure-time exercise until complete recovery, with return to activity advised after 30 days to 3 months, depending on disease severity.^7^ Similarly, the American Heart Association and American College of Cardiology (AHA/ACC) (2015) discourage competitive sports during acute pericarditis but recommend full activity only in the absence of disease evidence, without specifying timing or stratifying physical activity for athletes vs. non-athletes.^8,9^ However, these recommendations are primarily based on expert opinion rather than clinical trial evidence.

Complications linked to physical activity during active pericarditis are thought to involve immune-mediated mechanisms, supported by insights from animal models and autopsy studies, though much of the data pertains to myocarditis.^10,11^ Genetic variations of immune mediators, such as TNF polymorphisms, can predispose individuals to worse inflammation triggered by exercise.^11^ Expert experience has noted that patients receiving medical therapy for pericarditis who continue to exercise show worsening late gadolinium enhancement (LGE) on cardiac MRI, which tends to improve with exercise restriction. This was observed in our patient, whose first MRI, performed after four years of intermittent treatment, still showed moderate LGE.^12^ Current guidelines recommend colchicine and NSAIDs as first-line therapy for recurrent pericarditis, with corticosteroids reserved for refractory cases or contraindications to NSAIDs.^13^However, in patients with disease refractory to these therapies, biologics such as rilonacept, anakinra, and goflikicept have shown efficacy by targeting the IL-1 inflammatory pathway.^14^

In this patient, biologic therapy was initiated due to the refractory nature of his disease and the significant impact on his quality of life. Rilonacept reduces recurrence rates and improves outcomes in patients with recurrent pericarditis. It serves as the treatment of choice in refractory cases or in patients with contraindications to prolonged steroid use. However, it is unclear whether exercise can be safely resumed after 3–6 months of biologic therapy, necessitating individualized follow-up and monitoring. A recent review suggests a possible role for exercise testing prior to resumption.^12^

## Conclusion

Recurrent pericarditis triggered by exercise is an underrecognized phenomenon requiring a tailored, multidisciplinary approach. Biologics like rilonacept offer promising options for refractory cases, particularly when standard therapies fail to control symptoms. Early recognition of triggers, such as high-intensity exercise, and individualized treatment plans can optimize outcomes and improve quality of life for patients with recurrent pericarditis. Intense exercise can exacerbate recurrent pericarditis and should be considered a potential trigger.^1^ Biologics like rilonacept are valuable in managing refractory pericarditis and improving quality of life.^2,3^

Importantly, there is a lack of follow-up data on the efficacy and safety of rilonacept for exercise-induced pericarditis. However, its general safety and efficacy profile can be extrapolated from existing studies. Our patient did not experience any significant safety concerns with rilonacept, aside from mild injection site reactions that improved with steroids. Within 2 months of treatment, the patient was able to resume brisk walking and was off colchicine after one month.

## Lead author biography


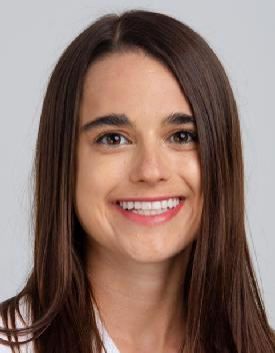


Tess Calcagno, PharmD, MD is a second-year internal medicine resident at Cleveland Clinic with aspirations in cardiology. Her academic interests include cardiac imaging, electrophysiology, exercise cardiology, and women’s cardiovascular health. She is actively involved in clinical research, including studies on myocardial volumes, mitral annular dysfunction, SGLT2 inhibitors in heart failure, and the interplay between exercise and cardiovascular health. Dr. Calcagno is dedicated to advancing patient care through evidence-based medicine and innovation, with a focus on optimizing outcomes in complex cardiovascular conditions. Outside of medicine, she enjoys long-distance running, exploring the outdoors, and promoting wellness among trainees.

## Data Availability

The data supporting the findings of this study are available within the article. Further details can be provided by the corresponding author upon reasonable request.

## Notes

**Consent:** Written informed consent was obtained from the patient for the publication of this case report, including all accompanying images and data. The authors confirm that written consent for submission and publication of this case report including images and associated text has been obtained the patient, in line with COPE guidance.

**Funding**: The authors have received no additional funding.

## References

[1] Andreis A, Imazio M, Casula M, Avondo S, Brucato A. Recurrent pericarditis: an update on diagnosis and management. Intern Emerg Med 2021;16:551–558. 33641044 10.1007/s11739-021-02639-6 PMC7914388

[2] Christou GA, Christou MA, Žiberna L, Christou KA. Indirect clinical markers for the detection of anabolic steroid abuse beyond the conventional doping control in athletes. Eur J Sport Sci 2019;19:1276–1286. 30880613 10.1080/17461391.2019.1587522

[3] Pelliccia A, Sharma S, Gati S, Bäck M, Börjesson M, Caselli S, et al 2020 ESC guidelines on sports cardiology and exercise in patients with cardiovascular disease. Eur Heart J 2021;42:17–96. 32860412 10.1093/eurheartj/ehaa605

[4] Cremer PC, Klein AL, Imazio M. Diagnosis, risk stratification, and treatment of pericarditis: a review. JAMA 2024;332:1090–1100. 39235771 10.1001/jama.2024.12935

[5] Maron BJ, Zipes DP, Kovacs RJ. Eligibility and disqualification recommendations for competitive athletes with cardiovascular abnormalities: preamble, principles, and general considerations: a scientific statement from the American Heart Association and American College of Cardiology. J Am Coll Cardiol 2015;66:2343–2349. 26542655 10.1016/j.jacc.2015.09.032

[6] Pelliccia A, Solberg EE, Papadakis M, Adami PE, Biffi A, Caselli S, et al Recommendations for participation in competitive and leisure time sport in athletes with cardiomyopathies, myocarditis, and pericarditis: position statement of the sport cardiology section of the European association of preventive cardiology (EAPC). Eur Heart J 2019;40:19–33. 30561613 10.1093/eurheartj/ehy730

[7] Pelliccia A, Sharma S, Gati S, Bäck M, Börjesson M, Caselli S, et al 2020 ESC guidelines on sports cardiology and exercise in patients with cardiovascular disease: the task force on sports cardiology and exercise in patients with cardiovascular disease of the European Society of Cardiology (ESC). Eur Heart J 2021;42:17–96. 32860412 10.1093/eurheartj/ehaa605

[8] Maron BJ, Udelson JE, Bonow RO, Nishimura RA, Ackerman MJ, Estes NM III, et al Eligibility and disqualification recommendations for competitive athletes with cardiovascular abnormalities: task force 3: hypertrophic cardiomyopathy, arrhythmogenic right ventricular cardiomyopathy and other cardiomyopathies, and myocarditis: a scientific statement from the American Heart Association and American College of Cardiology. Circulation 2015;132:e273–e280. 26621644 10.1161/CIR.0000000000000239

[9] Berglund F, Klein AL. Is exercise restriction necessary in patients with pericarditis? Cleve Clin J Med 2022;89:437–441. 35914934 10.3949/ccjm.89a.21120

[10] Grant JK, Shah NP. The impact of physical activity on pericarditis. Curr Cardiol Rep 2021;23:150. 34448954 10.1007/s11886-021-01578-0 PMC8390544

[11] Mackinnon LT . Immunity in athletes. Int J Sports Med 1997;18:S62–S68. 9129264 10.1055/s-2007-972701

[12] Shah NP, Verma BR, Ala CK, Khayata M, Phelan D, Imazio M, et al Exercise is good for the heart but not for the inflamed pericardium? JACC Cardiovasc Imaging 2019;12:1880–1881. 30878417 10.1016/j.jcmg.2019.01.022

[13] Adler Y, Charron P, Imazio M, Badano L, Barón-Esquivias G, Bogaert J, et al 2015 ESC guidelines for the diagnosis and management of pericardial diseases: the task force for the diagnosis and management of pericardial diseases of the European Society of Cardiology (ESC) endorsed by: the European association for cardio-thoracic surgery (EACTS). Eur Heart J 2015;36:2921–2964. 26320112 10.1093/eurheartj/ehv318 PMC7539677

[14] Klein AL, Lin D, Cremer PC, Nasir S, Luis SA, Abbate A, et al Efficacy and safety of rilonacept for recurrent pericarditis: results from a phase II clinical trial. Heart 2020;107:488–496. 33229362 10.1136/heartjnl-2020-317928 PMC7925818

